# Personal

**Published:** 1892-01

**Authors:** 


### Personal.

---

Dr. Nelson Saunders, of Randolph, N. Y., who has been ill for some weeks past, is reported convalescent, and his many friends will be glad to know that his recovery is confidently expected.

---

Dr. George Ben. Johnston, of Richmond, Va., was elected vice-president of the Southern Surgical and Gynecological Association, at its recent meeting, held in the capital of the Old Dominion. Dr. Johnston, who is a nephew of that distinguished soldier, Gen. Joseph E. Johnston, is one of the most genial and accomplished men of the South, and it was a fitting recognition of his ability to place him high on the list of officers of this excellent Association.

---

Dr. W. A. Putnam has been appointed surgeon of the Lake Shore R. R., at Westfield, *vice* Dr. Thomas D. Strong, resigned.

Personal.—Dr. Daniel Lewis, President of the New York Physicians' Mutual Aid Association, visited Buffalo in the latter days of December, on business connected with the useful institution which is receiving his most faithful and energetic service. During the two days he spent here much was accomplished in infusing a spirit of enthusiasm among the physicians of Buffalo, regarding this most noble charity. We hope to chronicle in a future number of the Journal some of the results of Dr. Lewis's timely visit.

